# Beyond the Screen: A Comprehensive Analysis of Emotional Skills and Social Networking in French Young Adults

**DOI:** 10.3390/ijerph21091176

**Published:** 2024-09-04

**Authors:** Cinzia Guarnaccia, Abdul Rahman Rasho, Benoit Testé, Sylvain Delouvée

**Affiliations:** Laboratoire de Psychologie: Cognition, Comportement, Communication (LP3C), Université Rennes 2, 35043 Rennes, France; abdul-rahman.rasho@univ-rennes2.fr (A.R.R.); benoit.teste@univ-rennes2.fr (B.T.); sylvain.delouvee@univ-rennes2.fr (S.D.)

**Keywords:** social networking, young adults, emotional intelligence, empathy, internet use, fear of missing out, mental health

## Abstract

(1) Background: This study investigates the influence of social networks on young adults, focusing on both positive and negative impacts. It considers problematic social networking site use (PSNSU), emotional intelligence, empathy, and the phenomenon of Fear of Missing Out (FoMO). (2) Methods: A comprehensive online survey was conducted with 442 participants aged 18 to 30 years that used various psychometric tools to assess emotional intelligence, empathy, internet and social media use, and FoMO. (3) Results: The study revealed that young adults display low competencies in emotional intelligence and empathy, with a tendency towards problematic internet and social media use. High FoMO scores were observed, correlating with negative internet use outcomes. Gender differences in these aspects were also explored. (4) Conclusions: The findings suggest a complex interaction between social network use, emotional skills, and FoMO that impacts young adults’ mental health and social behaviors. This study highlights the need for a nuanced understanding of these relationships and their implications for well-being and social interaction in the digital age.

## 1. Introduction

In recent years, social networks have become increasingly popular as an online activity. In 2020, social networking sites (SNSs) were used by 49% of the global population, tripling their total user base over the last decade [1]. This trend is particularly pronounced among young adults aged 18 to 24 years, with approximately 75% to 88% reporting at least one profile [2]. Social networking has emerged as a prevalent ‘media habit’ for many internet users [3], dominating modern life and influencing intergroup interactions and communication patterns [4], with positive impacts on well-being and social connection [5,6,7]. People in the post-modern world satisfy basic psychological needs through social network presence, including the need for belonging, social interaction, fulfillment, competence, and personal identity [8,9,10,11].

However, an increase in SNS use can lead to problematic social networking site use (PSNSU), affecting between 1.6% and 34% of various populations [12]. This issue is of particular concern for adolescents and young adults, with terms like abuse, addiction, or compulsion used to describe their use profiles, potentially leading to life-threatening consequences [13,14]. Findings on the impact of social networks on the physical and mental health of young adults are mixed, with some authors highlighting negative effects [15,16,17,18,19] while others emphasize positive aspects in terms of prevention and health promotion [20,21,22], especially during the COVID-19 pandemic years [23,24].

A variety of studies have identified several predictors of different social network use styles, including attachment [25,26], self-esteem [27,28], emotion recognition [29], as well as socio-emotional skills and executive functions in general [30,31]. Marín-López et al. [32] explored the relationships between social and emotional competencies, online emotional content, cybervictimization, and cyberperpetration among adolescents, finding that high levels of social and emotional competencies were negatively related to cybervictimization and cyberperpetration, and that excessive use of online emotional content was a risk factor for cyberbullying.

The relationship between emotional intelligence and problematic social network use has also been investigated, with some studies finding a significant association [33,34,35] and others suggesting more heterogeneous relationships indicating a moderating or mediating role of emotional intelligence [36,37,38]. Authors including Abu-Shanab and Abu-Shanab [39], Newness [40], Miller [41], and Morelli et al. [42] have found negative associations between emotional intelligence and PSNSU, predicting behaviors on social networks such as inappropriate posting, disclosure, and sexting.

Empathy has been studied as a factor impacting the style and quality of social network use [43,44,45]. Guan et al. [46] noted in their meta-analysis that social media use was positively related to affective empathy, but only marginally related to cognitive empathy, highlighting a ‘media-empathy paradox’, where a tool created for social connection may reduce connective capacities [46,47,48]. Researchers have emphasized the role of social media in increasing focus on oneself rather than others, potentially decreasing empathy and increasing more self-oriented personality traits (narcissism, self-satisfaction, etc.), with results varying depending on the type of online activity [49,50,51,52].

Special attention has been given to the innovative construct of FoMO (*Fear of Missing Out*). Defined as the ‘desire to stay continually connected with what others are doing’ [53], FoMO has been associated with a range of negative life experiences and feelings and found to be inversely proportional to life satisfaction [53,54,55]. It is linked to social media use and addiction [54,56,57,58]. Fioravanti et al. [59] conducted a meta-analysis confirming the robust association between FoMO levels and SNS use and PSNSU. Aliverdi et al. [60] studied the effect of social networks and online emotional relationships on students’ mental health and quality of life, finding that internet use, virtual networks, online emotional relationships, and unfavorable socioeconomic status were associated with mental disorders and reduced quality of life in students. Hunt and Krishnan [61] assessed how attitudes towards social networks and their use influence emotional well-being, discovering that social network use negatively impacted users’ emotional well-being.

A recent study [62] on university students in Türkiye revealed that procrastination mediates the relationship between the Fear of Missing Out (FoMO) and internet addiction. FoMO directly increases both procrastination and internet addiction, with procrastination further exacerbating internet addiction. Addressing procrastination behaviors can thus be crucial in interventions aimed at reducing internet addiction driven by FoMO. A study among Italian adolescents found that females reported higher levels of compulsive internet use and alexithymia compared to males, while dissociation levels were similar [63]. Dissociation significantly mediated the relationship between alexithymia and compulsive internet use, particularly in females. Another line of research has investigated the link between social network use (and problematic use) and antisocial behaviors, particularly in younger generations [64,65,66]. FoMO appears to be an additional risk factor for (especially cyber) victimization in adolescence [67,68,69].

This study examines the intricate relationship between socio-emotional competencies and behaviors of aggression and victimization among young adults. Focusing on emotional intelligence, empathy, FoMO, and PSNSU, it aims to explore how these factors interrelate with the risk of becoming a perpetrator or victim. We hypothesize that in our sample of young adults, problematic behaviors in internet and social network usage are associated with lower social–emotional competencies (such as emotional intelligence and empathy) in “offline” life (H1). Additionally, the study investigates whether FoMO mediates this relationship (H2), considering various aspects such as gender differences and the impact of socio-emotional skills on online behaviors. This research seeks to offer a comprehensive understanding of these dynamics and their implications for mental health and digital interaction among youth.

## 2. Materials and Methods

### 2.1. Participants and Procedures

Participants were recruited via online panels using CrowdPanel. Our target sample size was 500 participants, but, anticipating exclusions, we committed to recruiting 600 participants. The only inclusion criteria were nationality (French) and age (between 18 and 25 years). Recruitment took place between 11 July 2023 and 30 June 2024. The study was published online via Limesurvey (Version 5.6.11+230320) and distributed by CrowdPanel. It took approximately 15 min to complete. In accordance with ethical and deontological rules (see Ethics Statement at the end), participants in our research were volunteers and gave their informed consent. The data were processed anonymously.

In total, 549 participants completed our questionnaire. A total of 15 participants were excluded based on an attention check, 80 participants were excluded for partial and incomplete answers, and 12 participants were excluded because they were underage. This left us with 442 participants: 302 (68.3%) identified as female, 131 (29.6%) identified as male, and 9 (2%) identified as non-binary gender (other). The age range was from 18 to 30 years, and the average age of the participants was 22 years (M = 22.6, SD = 4.73).

In terms of participants’ level of education, 7.7% of the sample had obtained a secondary school leaving certificate, 36% of the sample had two years of higher education, 33.5% had three years of higher education, and 22.6% had five years of higher education. With regard to occupation, 61.66% (N = 271) of the sample were students, followed by 18.1% (N = 80) employees and, to a lesser extent, other occupations.

### 2.2. Measures

The Profile of Emotional Competence (PEC, [70]) was used to measure Emotional Intelligence (EI). The PEC questionnaire, in the original French form (50 items), measures intrapersonal and interpersonal EI separately by five core emotional competences distinctly for one’s emotions and others’ emotions. The questionnaire encompasses 10 subscales (intrapersonal identification, intrapersonal expression, intrapersonal comprehension, intrapersonal regulation, intrapersonal utilization, interpersonal identification, interpersonal expression, interpersonal comprehension, interpersonal regulation, and interpersonal utilization) of 5 items each (with 2 or 3 reverse items) grouped into two factors (intrapersonal EC and interpersonal EC) and one total score.

The instructions ask participants to answer questions “to better understand how they live with their emotions” (example of an item: “*My emotions appear without me understanding where they come from*”; “*It’s difficult for me to explain to others what I’m feeling even if I want to*”). Each participant rates their degree of agreement with the proposed statements on a Likert scale from 1 to 5 (1 means that the statement does not correspond to you at all or that you never react in this way; on the contrary, 5 means that you recognize yourself completely in what is described or that it happens to you very often).

The validation study suggests that the questionnaire has good psychometric properties with a good internal consistency for the two global scores (interpersonal EC and intrapersonal EC) as well as the total score. In our study, we obtained excellent Cronbach alpha values (α = 0.90 for the total scale, α = 0.87 for the interpersonal EC scale, and α = 0.86 for the intrapersonal EC scale), thus confirming the good internal consistency of the instrument for our data.

The Interpersonal Reactivity Index (IRI, [71,72]), a 28-item self-report questionnaire, was used to measure four different dimensions of dispositional empathy. These subscales (each made up of 7 different items) are Empathic Empathy (emotional empathy), Perspective Taking (cognitive empathy), Fantasy (empathy for fictional characters), and Personal Distress (self-focused responses to others’ suffering). In this study, we used the French short version from Brown et al. [73] which consists of 15 items. It uses a 5-item Likert scale with two anchors (A = Does not describe me well; E = Describes me very well) that can be summed (using 1 to 5 endpoints). The IRI is a continuous measure of empathy in normal populations and not a categorical measure (“high empathy” versus “low empathy”); each subscale should be used separately since the instrument is not intended to measure global empathy. The psychometric properties of the IRI are attested by several studies; in our sample, we found good internal consistency values for all subscales (Cronbach alpha between 0.62 and 0.80).

The Generalized Problematic Internet Use Scale 2 (GPIUS2, [74]; French version [75]) was used to assess problematic internet use. The GPIUS2 is a self-administered questionnaire that evaluates problematic internet use (PIU) from a multidimensional perspective based on Davis and Caplan’s cognitive–behavioral model of generalized PIU [76,77]. The GPIUS2 measures four constructs linked to PIU: (1) Preference for Online Social Interaction (POSI), (2) mood regulation, (3) deficient self-regulation (consisting of a compulsive use subscale and a cognitive preoccupation subscale), and (4) negative outcomes. Participants were instructed to rate the extent to which they agreed with each of the 15 GPIUS2 items on a scale ranging from 1 (‘‘definitely disagree”) to 8 (‘‘definitely agree”). In the original validation study, the overall reliability of the composite GPIUS2 (where all 15 scale items are included) was alpha = 0.91 and 0.88 for the French version (ranging between 0.66 and 0.83 for the subscales); in our study we found a good internal consistency with an overall alpha = 0.84.

The Bergen Social Media Addiction Scale (BSMAS, [78], French version by [79]) was used to specifically assess the problematic use of social media. The 6-item scale is adapted from the Bergen Facebook Addiction Scale [78] and measures addiction behaviors, and is rated on a five-point Likert scale ranging from 1 (very rarely) to 5 (very often). The items/indicators assess specific addiction facets: (1) salience, (2) tolerance, (3) mood modification, (4) relapse/loss of control, (5) withdrawal, and 6) conflict/functional impairment.

Previous studies have proposed a cut-off of 19 [80] or 24 [81] to classify an adolescent as suffering from Problematic Use of Social Media (based on DSM 5 criteria); however, these studies are always based on samples of a younger age than our study. We also consider here the criterion initially proposed by Andreassen et al. [78], indicating that a score above 3 for at least 4 out of 6 items can be considered as an indicator of addiction. Internal consistency of the scale was very good both in the validation sample (alpha = 0.84) and in our sample (alpha = 0.82).

The Fear of Missing Out scale (FoMOs, [53]) was used to assess the degree to which one fears missing out on social events, in particular involving their friends, and often using social media to stay (hyper) connected. As reported by Bowman and Gordon [82], the fear of missing out has been cited as a motivation for using Facebook [83] as well as a reason for the emergence of separation anxieties between smartphone users and their smartphones [84]; consequently, the FoMO scale may be useful across many technologically mediated communication contexts.

The final version of this tool is a 10-item unidimensional scale set on 5-point Likert-type responses. Individual scores can be computed by averaging responses to all ten items with good internal consistency (alpha = 0.87 in the validation study and 0.76 in our sample).

We also questioned the participants about their experiences of violence, either as perpetrators or as victims. The experience of perpetration was questioned by means of a question on insulting behavior (sexist, racist, homophobic, etc.), moral harassment, assault, and physical or sexual violence that the person may have committed in the last 12 months. The victim’s experience was addressed by means of a question asking them to specify experiences of insults or threats (sexist, homophobic, etc.), stalking, and physical and sexual aggression in the last 12 months. For the most intense experience (or for the last one in chronological order), the person could specify some elements related to his or her experience (behaviors, emotions, etc.) and the solutions he or she had chosen to resort to in order to react to the situation.

Finally, we asked the participants for some socio-demographic information (gender, age, level of education, and employment status).

### 2.3. Data Analysis

We conducted a correlational and cross-sectional study. Data analysis was carried out using Jamovi (version 2.3.17) software. We tested the normality of the distribution of all our variables by calculating the skewness and kurtosis coefficients. The internal coefficient of the measures used was tested by calculating the Cronbach’s alpha coefficient. We carried out descriptive statistics and tested the differences between independent groups to assess the distribution of the variables in our sample. Correlation analyses (Pearson’s R) were carried out to calculate the associations between variables in our sample. In order to test the hypothesized mediation effect, we used bootstrapping with 5000 randomly generated samples to test the significance of indirect effects [85]. Standardized coefficients (β) and statistical significance are reported for each regression equation. Because our model was a just-identified model with zero degrees of freedom, we did not compute model fit indices [86].

## 3. Results

### 3.1. Socio-Emotional Competences

In the first section of the questionnaire, participants answered questions from the PEC and the IRI with the aim of assessing their social–emotional skills in Emotional Intelligence and Empathy.

With respect to Emotional Intelligence, the results show (Table 1) a sufficiently good profile, albeit with significantly lower averages than the authors found in the validation study (with a total score mean of 3.29 compared with a mean of 3.37, *t* = −3.55, *p* < 0.001). The participants in our study have low scores in all the above variables measuring interpersonal skills, and, in particular, men appear to perform poorer in interpersonal emotional intelligence, indicating that they are less skilled in understanding and expressing emotions in relationships with others. On the other hand, women appear to be less skilled in the internal regulation of their emotions.

With respect to empathy, the participants in our study obtained lower scores than the validation study; in particular, significantly lower scores were found with respect to the Fantasy (mean of 15.2 compared to a normative value of 17.04, *t* = −10.3, *p* < *0*.001), Perspective Taking (mean of 14.7 compared to a normative value of 16.77, *t* = −13.1, *p* < 0.001), Personal Distress (mean of 11.8 compared to a normative value of 12.08, *t* = −3.22, *p* = 0.001), and Empathic Concern subscales (mean of 11.3 compared to a normative value of 19.07, *t* = −67.9, *p* < 0.001). Furthermore, in our sample (Table 1), men scored significantly lower than women on the Fantasy and Empathic Concern subscales.

### 3.2. Internet and Social Media Use

In the second part of this study, we attempted to measure the participants’ habits with regard to the use of social networking sites, with the aim of identifying any problematic use and to understand what patterns were most frequent among the young participants in the study. For this purpose, we administered the GPIUS2 and the BSMAS scales.

The results show (Table 2) that the Mood Regulation and the Deficient Self-Regulation subscales obtained the highest scores, highlighting the presence of problematic behaviors with respect to the use of the internet as a tool to regulate emotions in relationships with others (e.g., using the internet to talk to others when feeling lonely, angry, or down), with the presence of a deficient self-regulation, and with cognitive preoccupation and compulsive internet use (with obsessive thoughts, difficulty in controlling the time spent online, and the urgency to connect when offline). The mean score obtained on the BSMAS (Table 2) does not indicate addictive behavior in relation to the use of social media according to the cut-offs used for the instrument. However, 117 participants (26.47% of our sample) scored above the cut-off of 19 and exceeded the threshold of four items with a score above 3; therefore, we can consider that they exhibit addictive behavior with respect to their social media use. No significant gender differences were found in our sample concerning these behaviors.

### 3.3. Fear of Missing Out

Finally, we measured the Fear of Missing Out levels of the participants in our study by means of the FoMO scale. Compared to the sample used in study 3 of the validation article (similar in age to ours), our participants obtained significantly higher FoMO scores (mean of 2.77 compared to 2.37, *t* = 10.8, *p* < 0.001). We did not detect any gender differences with respect to this variable.

### 3.4. Victimization or Perpetration Experiences

Among the questions asked, our participants were asked whether in the last twelve months they had been victims of (1) insults, threats of a sexist, racist, or homophobic nature, etc.; (2) harassment in a training, work, or leisure context; (3) physical aggression; and (4) sexual aggression. The testimonies obtained highlight the complexity and variability of the impact of aggression or victimization on individuals in different social and personal contexts (Table 3 and Table 4).

Some participants reported having encountered none of the events listed, while others were unable to recall specific incidents. This phenomenon could be attributed to psychological defense mechanisms such as repression, or to a genuine absence of victimization experience. Table 3 shows the percentage of participants by gender who had at least one experience of victimization. While insults and threats are the most common, it should be noted that all forms of victimization are present in our sample.

Verbal abuse was predominant in the accounts, with incidents ranging from family arguments where shouting was exchanged to misogynistic insults at public events. For example, one individual described an episode of verbal abuse during a conflict with a relative, characterized by extreme alertness and ongoing stress as consequences. Another testimony mentions, *“I think the last time it happened was when I was at school, when I was often the target of gratuitous insults from my classmates.”* Online harassment was also mentioned, illustrated by a case where a person received derogatory comments on dating apps, suggesting unsolicited and degrading hypersexualization. The experience was particularly humiliating.

Incidents of physical harassment were reported in public spaces, such as bars and streets, where individuals were inappropriately touched or prevented from leaving. One victim recounted a situation in which she was forcibly restrained by a man who refused to let her leave despite her repeated protests, an experience that seems to have resulted in an oppressive sense of helplessness and danger. A respondent shares, *“I was assaulted in a bar, a drunken man grabbed me and wouldn’t let me go despite my repeated protests.”* School harassment is another facet of victimization, with accounts of severe verbal abuse, racial insults, and mockery, often accompanied by physical assaults. One testimonial reveals the painful reality of a participant who, at 15 years old, was subjected to degrading insults and physical violence, culminating in an act in which his clothes were torn.

Experiences of physical violence include assaults in public places, with victims reporting being beaten by intoxicated individuals, snatched, or sexually assaulted. For example, one person described being followed and physically assaulted, leading to a persistent fear of frequenting certain urban areas. A participant narrates, *“In the days that followed, I was afraid of running into them again and I avoided certain places for fear of reliving the attack.”* Examples of mobbing at work were also reported, where individuals were confronted with insults and threats, often from disgruntled customers or colleagues. One particularly troubling case involved an individual harassed by a customer, resulting in a chronic sense of insecurity and a generalized distrust of certain professional interactions. Another account states, *“I was the victim of harassment at work, repeated insults from a disgruntled customer, which made me very suspicious in my professional interactions.”*

The collected responses highlight the adverse consequences of victimization, which range from anxiety and fear to changes in daily behaviors, such as avoiding certain places or adopting increased safety measures. Coping strategies mentioned include ignoring the incidents, support from family and friends, psychological counselling, and, in some cases, the mobilization of legal or administrative recourses. These stories underline the urgency of approaching victimization prevention and support from a holistic, multidimensional perspective.

A recent study among Italian high school students revealed that high levels of emotional regulation, attention impulsiveness, online vigilance, and multitasking are significantly linked to increased internet addiction [87]. Adolescents with emotional and behavioral problems, especially those exhibiting ADHD symptoms, are particularly vulnerable to problematic smartphone use. These findings emphasize the need for targeted screening and intervention strategies.

In the experiences of victims, stories also reveal varying degrees of emotional impact, from those who experienced no noticeable trauma to individuals who seem deeply affected by their experiences. A significant example is that of a person who had been harassed in the street who described a lasting feeling of oppression and insecurity. Recourse varies according to the severity of the incident and the individual’s resilience. Some chose not to respond or to ignore the aggression, while others sought support from family, friends, or professionals. An illustrative case is that of a person who called on security to resolve a confrontation, demonstrating a proactive approach in the face of adversity. While some testimonies reflect an ability to overcome the experience, others underline the need for external recourse to ensure the victim’s recovery and safety. Another respondent reflects, *“I was aware that my fear was irrational, but it led me to avoid certain situations and to be more vigilant”*.

### 3.5. Correlations

**Figure 1:**
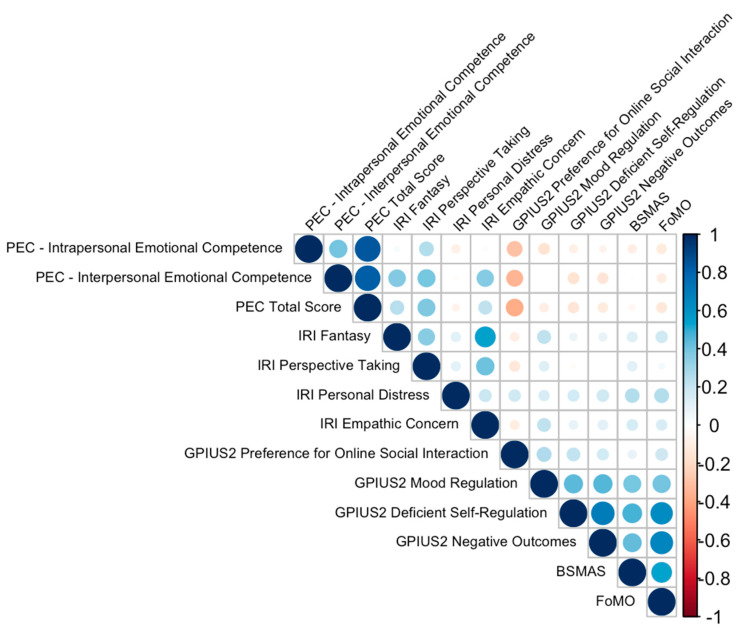
Correlation matrix.

An analysis of the correlations between our different variables (see Figure 1) yielded some interesting results. There was obviously a strong significant positive correlation (*r* = 0.407, *p* < 0.001) between the two skill domains (interpersonal and intrapersonal). This means that individuals with good emotional skills in one domain tend to have good emotional skills in the other domain. With regard to empathy, the ability to adopt the perspective of others seems to be associated with the tendency to identify with fictional characters (*r* = 0.356, *p* < 0.001). There was a significant positive correlation between the interpersonal emotional competences and most of the IRI’s subscales (Fantasy: *r* = 0.375, *p* < 0.001; Perspective Taking: *r* = 0.385, *p* < 0.001; Empathic Concern: *r* = 0.363, *p* < 0.001).

Finally, with regard to problematic internet use, significant relationships were observed between different problematic aspects, including a preference for online social interaction, deficient self-regulation, and negative outcomes. Firstly, there was a significant correlation (*r* = 0.263, *p* < 0.001) between a preference for online social interaction and mood regulation. In other words, people who tend to prefer online social interaction tend to have less effective mood regulation. Secondly, there was a highly significant positive correlation (*r* = 0.493, *p* < 0.001) between deficient self-regulation with internet use and mood regulation. This suggests that these aspects of problematic internet use are closely related. Thirdly, and even more interestingly, there was a highly significant positive correlation between problematic internet use scores (all GPIUS subscales and BSMAS score) and fear of missing out (FoMO) on social events. This suggests that those with negative internet use outcomes also tend to experience more fear of missing out on social events.

### 3.6. Mediation Model

**Figure 2:**
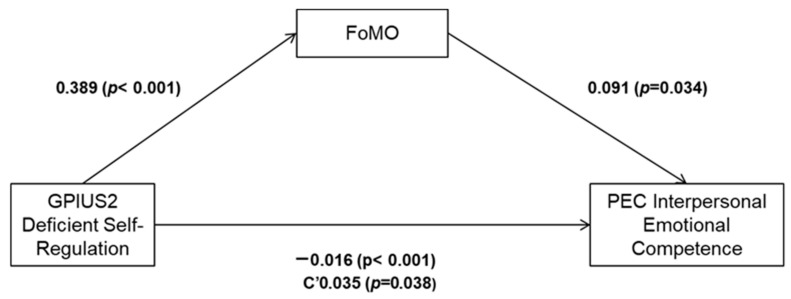
Mediation Model.

Consistent with the correlation analysis and according to the articulation of our research, we tested our last hypothesis by using a mediation model to explore the relationships between the Deficient Self-Regulation subscale of GPIUS, FoMO, and Interpersonal Emotional Competence. Analysis of the mediation model showed that the effect of Deficient Self-Regulation in Generalized Problematic Internet Use on Interpersonal Emotional Competence (offline) was mediated by FoMO. As the standardized regression coefficients for the direct effects of the variables show (Table 5, Figure 2), the GPIUS Deficient Self-Regulation was significantly related to FoMO and Interpersonal Emotional Competence. FoMO was also significantly linked to Interpersonal Emotional Competence.

Results of the bootstrapping analysis (Table 6) showed that FoMO partially mediated the effect of Deficient Self-Regulation in Generalized Problematic Internet Use on Interpersonal Emotional Competence via significant direct and indirect effects.

## 4. Discussion

The main aim of this cross-sectional study was to investigate emotional intelligence, empathy, FoMO, and PSNSU, and also to explore how these factors interrelate with the risk of becoming a perpetrator or victim in young adults. Our results showed several aspects that, although they need further investigation, seem interesting to emphasize.

Firstly, in line with the literature on the topic, we found low levels of Emotional Intelligence [88,89] and Empathy [46,90,91] in our sample of young French adults. Young people, especially men, displayed poor interpersonal skills and low levels of empathy, and seemed to experience particular difficulties in their relationships with others. This appears to be a source of vulnerability during a developmental phase where, having overcome adolescence and its identity reorganizations, there should be a focus on consolidating a healthy relational style. Their low levels of empathy, both emotional and cognitive, expose them to the risk of not truly ‘meeting’ the other in a relationship but ‘experiencing’ relationships where the other is merely a means to fulfill their own needs.

Rather than stabilizing their identity, they seem to navigate in what Ferraro calls ‘disidentity’, a phenomenon characterizing the existential condition of young adults struggling to realize their existential project and themselves. In more in detail, the neologism disidentity [92,93] highlights the condition of indefiniteness, emptiness, lack of plans, etc., corresponding to the breakdown of existential and cultural matrices observed in the post-modern world, thereby presiding over the emergence of new forms of identity, sociality, and potentially psychological suffering [94]. In this context of vulnerability, young people in our sample appear to use the internet as an ‘appendix to the Self’, regulating their emotions and interacting with others in a way perceived as safer than offline relationships. Moreover, we observed high levels of FoMO, identified in the literature as a significant predictor of problematic social media use [59,60,95]. In this context, the frequencies of violent experiences as victims or perpetrators are particularly worrying, suggesting a link between poor emotional skills, the use of ‘relationship mediators’ such as social networks [25,29], and the risk of violent behavior [64,65,66].

The mediation analysis in our study sheds light on the intricate relationships between emotional competencies, FoMO, and problematic internet use. Notably, the findings indicate a partial mediation effect of FoMO on the link between emotional competencies and internet use behaviors. This suggests a complex interdependency where lower emotional skills may increase FoMO, potentially leading to more problematic internet use. These insights contribute to the understanding of young adults’ digital engagement, highlighting the nuanced role of emotional competencies in the context of digital communication. Our results, in line with those reported in [96], emphasize the importance of interventions focusing on social–emotional skills to prevent problematic behavior related to the use of social networking sites.

This study, while contributing valuable insights into the socio-emotional dynamics of young adults in digital environments, presents certain limitations that merit consideration. The sample size, although statistically sufficient, may not encompass the wide array of socio-demographic backgrounds necessary for a more comprehensive understanding of the studied phenomena. This limitation restricts the generalizability of the findings to a broader population. Additionally, the cross-sectional design of the study precludes the ability to draw causal inferences or observe the evolution of behaviors and attitudes over time, which could be pivotal in understanding the progression of emotional intelligence and social media use. Furthermore, the analysis of victimization experiences requires a more nuanced approach. The current methodology does not delve deeply into the complexities and varied contexts of these experiences, potentially overlooking critical aspects of victimization in digital spaces. Future research, ideally employing longitudinal designs and a more diverse sample, could significantly enhance our understanding by providing a more detailed and dynamic picture of these interactions.

## 5. Conclusions

In conclusion, this study underscores the vulnerability of young adults in a digital context, particularly highlighting the risks of isolation and entrapment in virtual environments. It draws attention to the necessity of understanding online relationships as reflections and extensions of real-life social interactions. The findings suggest a potential heightened risk of resorting to violence due to emotional and empathetic deficits, especially among males who are part of a generation where emotions are central yet poorly managed. This research points towards the importance of fostering genuine, reciprocal relationships in digital spaces, countering tendencies towards narcissistic needs fulfillment. These insights call for further exploration, possibly extending to adult samples, and also examining other variables such as attachment styles in digital interactions to better understand and mitigate these vulnerabilities.

## Figures and Tables

**Table 1:** Gender differences in Emotional Intelligence and Empathy among young adults.

	Gender	N	Mean	SD	F	*p*	Post Hoc Games–Howell Test
PEC—Intrapersonal identification of emotions	Women	302	3.23	0.852	3.585	0.045	Women < Men
	Men	131	3.42	0.683			
	Other	9	2.98	0.919			
PEC—Interpersonal identification of emotions	Women	302	3.83	0.729	18.323	<0.001	Men < Women
	Men	131	3.35	0.751			
	Other	9	3.78	1.074			
PEC—Intrapersonal comprehension of emotions	Women	302	2.98	0.897	2.993	0.071	-
	Men	131	3.18	0.698			
	Other	9	3.18	1.007			
PEC—Interpersonal comprehension of emotions	Women	302	3.72	0.674	18.356	<0.001	Men < Women
	Men	131	3.27	0.724			
	Other	9	3.96	0.979			
PEC—Intrapersonal expression of emotions	Women	302	3.02	0.863	0.818	0.455	-
	Men	131	3.04	0.726			
	Other	9	2.67	0.831			
PEC—Interpersonal expression of emotions	Women	302	4.06	0.732	27.982	<0.001	Men < Women
	Men	131	3.44	0.811			
	Other	9	3.89	0.889			
PEC—Intrapersonal regulation of emotions	Women	302	2.65	0.853	15.449	<0.001	Women < Men
	Men	131	3.11	0.787			
	Other	9	2.40	0.775			
PEC—Interpersonal regulation of emotions	Women	302	3.25	0.670	1.765	0.195	-
	Men	131	3.11	0.754			
	Other	9	3.38	0.644			
PEC—Intrapersonal utilization of emotions	Women	302	3.56	0.729	6.935	0.005	Men < Women
	Men	131	3.29	0.696			
	Other	9	3.69	0.679			
PEC—Interpersonal utilization of emotions	Women	302	2.91	0.824	0.514	0.605	-
	Men	131	2.93	0.862			
	Other	9	3.33	1.261			
PEC—Intrapersonal emotional competence	Women	302	3.09	0.615	2.601	0.097	-
	Men	131	3.21	0.493			
	Other	9	2.98	0.493			
PEC—Interpersonal emotional competence	Women	302	3.55	0.495	15.923	<0.001	Men < Women
	Men	131	3.22	0.585			
	Other	9	3.67	0.752			
PEC total score	Women	302	3.32	0.466	2.260	0.129	-
	Men	131	3.21	0.480			
	Other	9	3.32	0.608			
IRI Fantasy	Women	302	15.81	3.681	14.587	<0.001	Men < Women
	Men	131	13.70	3.689			
	Other	9	14.44	4.927			
IRI Perspective Taking	Women	302	14.85	3.293	1.031	0.374	-
	Men	131	14.43	3.194			
	Other	9	15.56	3.358			
IRI Personal Distress	Women	302	11.65	1.954	2.143	0.141	-
	Men	131	12.08	2.012			
	Other	9	11.78	1.302			
IRI Empathic Concern	Women	302	11.69	2.277	11.471	<0.001	Men < Women
	Men	131	10.47	2.447			
	Other	9	11.56	2.789			

**Table 2:** Means and standard deviations of internet and social media use among young adults.

	Mean	SD
GPIUS2 Preference for Online Social Interaction	2.27	1.18
GPIUS2 Mood Regulation	3.43	1.10
GPIUS2 Deficient Self-Regulation	2.86	0.981
GPIUS2 Negative Outcomes	2.04	0.996
BSMAS	15.0	5.66

**Table 3:** Prevalence of Victim Experiences Among Participants by Gender.

Gender	Insult Victim	Harassment Victim	Physical Aggression Victim	Sexual Aggression Victim
Other	1.5%	0.5%	0	0.2%
Women	33.5%	8.8%	7.2%	7.2%
Men	15.6%	5.7%	5%	2.5%

**Table 4:** Incidence of Perpetrator Behaviors Among Participants by Gender.

Gender	Insult Perpetrator	Harassment Perpetrator	Physical Aggression Perpetrator	Sexual Aggression Perpetrator
Other	0.2%	0	0	0
Women	16.3%	7.2%	4.3%	3.8%
Men	7.9%	4.3%	3.2%	1.4%

**Table 5:** The Mediating Role of FoMO in Internet Use and Social Skills.

			Bootstrapping Bias 95% Confidence Interval			
Effect	Estimate	SE	Lower	Upper	Z	*p*
Indirect	0.035	0.017	0.002	0.0694	2.08	0.038
Direct	−0.2126	0.032	−0.189	−0.061	−3.87	<0.001
Total	−0.091	0.026	−0.141	−0.038	−3.46	<0.001

**Table 6:** Bootstrapping Analysis of Emotional Competence, FoMO, and Internet Use.

					95% Confidence Interval			
			Estimate	SE	Lower	Upper	Z	*p*
GPIUS2 Deficient Self-Regulation	→	FOMO_TOT	0.389	0.033	0.324	0.454	11.71	<0.001
FOMO_TOT	→	PEC—Interpersonal Emotional Competence	0.091	0.0432	0.005	0.175	2.12	0 .034
GPIUS2 Deficient Self-Regulation	→	PEC—Interpersonal Emotional Competence	−0.126	0.326	−0.189	−0.0617	−3.87	<0.001

## Data Availability

All study materials, data, and analysis codes are available on OSF (https://osf.io/7d4fa/?view_only=3ee110d120f9420a9965d7b30f8913a1, accessed on 31 August 2024).
